# Mixture to Prevent Toothache

**Published:** 1888-11

**Authors:** 


Mixture to Prevent Toothache.—Rub the gums with the following mixture:

| | |
|---|---|
| ℞. Hydrochlorate of cocaine - - - | 1 gr. 25 |
| Hydrochlorate of morphia - - - | 0 gr. 30 |
| Benzoic acid - - - - - - | 9 gr. 40 |
| Eugenol - - - - - - - | 3 gr. 75 |
| Absolute alcohol - - - - | 30 gr. |

(*Revue de Therapeutique.*)

